# Editorial: Optimization strategies for pain management with neuromodulation

**DOI:** 10.3389/fpain.2022.1012790

**Published:** 2022-09-15

**Authors:** Kevin Pacheco-Barrios, Sandra Carvalho, Jorge Leite, Wolnei Caumo, Felipe Fregni

**Affiliations:** ^1^ Neuromodulation Center and Center for Clinical Research Learning, Spaulding Rehabilitation Hospital, Massachusetts General Hospital, Harvard Medical School, Boston, MA, United States; ^2^ Vicerrectorado de Investigacion, Unidad de Investigacion para la Generacion y Síntesis de Evidencias en Salud, Universidad San Ignacio de Loyola, Lima, Peru; ^3^ Translational Neuropsychology Lab, Department of Education and Psychology, University of Aveiro, Aveiro, Portugal; ^4^ Portucalense Institute for Human Development, INPP, Rua Dr. António Bernardino Almeida, Portucalense University, Porto, Portugal; ^5^ Pain and Palliative Care Service, Laboratory of Pain and Neuromodulation, Hospital de Clínicas de Porto Alegre, Porto Alegre, Brazil

**Keywords:** neuromodulation, chronic pain, biomarkers, pain management, tDCS – transcranial direct current stimulation

**Editorial on the Research Topic** Optimization strategies for pain management with neuromodulation

Chronic pain is a high-priority global health issue due to its high prevalence, impact on quality of life, and cost (1). In most cases, chronic pain is challenging to manage, and the existing treatment modalities have reported frequent and severe adverse events, including gastritis (2), cardiovascular complications (2), or even addiction (1) in the case of opioids. Although, during the past two decades, neuroscientific studies have increased our understanding of the pain experience as a complex individual multidimensional phenomenon, the current widespread management methods are still ignoring this nature. Moreover, due to geographical and socioeconomic barriers, there is high inequity in pain treatment access (3). Therefore, chronic pain management urgently requires innovative approaches to shift the target of interventions and modify the “delivery model” from an in-person only provider-centered system to a hybrid patient-centered model (4, 5).

Neuromodulatory interventions are promising management options that target maladaptive neuroplasticity, which has been associated with chronic pain conditions (6). Techniques such as transcranial direct current stimulation (tDCS) and repetitive transcranial magnetic stimulation (rTMS) have shown adequate efficacy and safety profiles (7). However, recent meta-analyses have reported a high within-and between-study variability and mixed effect sizes, hindering their implementation in clinical practice (7). Additionally, small sample sizes, parameters variability (6), and also lack of device portability and limited easy-to-use profile reduce its competence and applicability compared to “standard” pain treatments. Indeed, one of the main issues in this field is that the alternative, pharmacological treatments, is very easy to use (taking a pill is something very quick) and has a large immediate effect size (compared to neuromodulation which may take several sessions to have an effect).

Under this scenario, one potential solution is to systematically optimize these interventions, including treatment protocols, biomarkers, and delivery models, inducing a shift from a pathway of “sustaining innovation” to “disruptive innovation” (8). According to Christensen's theory (8), disruptive innovation defines a process by which an enterprise, product, or service initially takes root in simple applications in an overlooked sector of the market—usually by being effective, safer, affordable, and accessible—and then persistently moves upmarket, eventually displacing established products. This approach does not necessarily require a breakthrough technology but a strategic (and creative) use of technology and user-centered design (8). The principles of disruption theory can be easily applied to pain neuromodulation to finally harness its potential therapeutic applications.

**Figure 1:**
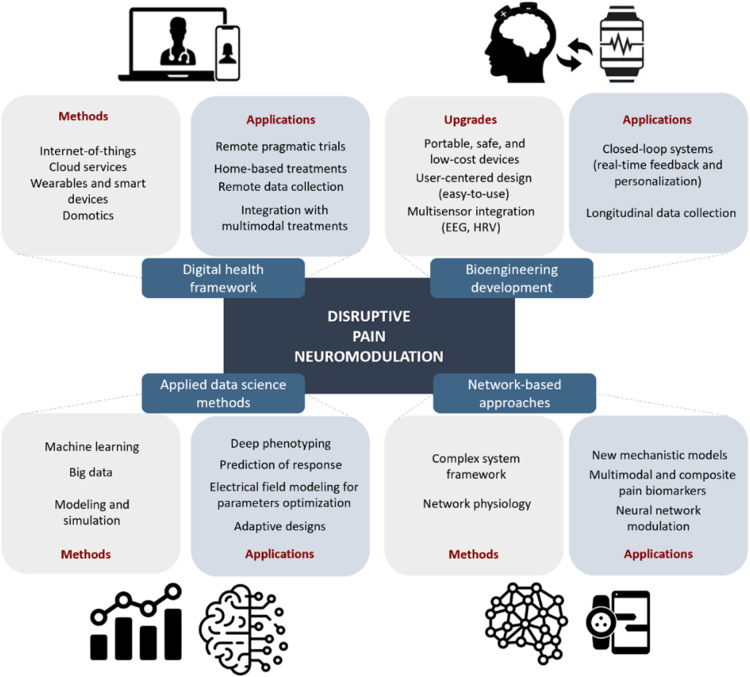
Development areas for disruptive pain neuromodulation therapies.

This research topic aimed to gather original research and reviews illustrating the recent advances in this optimization pathway. To provide a broader context for the studies, we propose a framework for “disruptive pain neuromodulation” (Figure 1), where we underscore four optimization domains that require development: (1) Digital health framework (9)—the inclusion of these methodological tools will allow home-based interventions and remote trials targeting populations that are usually excluded or not receiving pain management (the overlooked sector of the “market”). (2) Bioengineering development (10)—which will provide portable, safe, and low-cost devices, including user-centered designs with the potential of closed-loop and easy-to-use systems (the “convenient” and “affordable” product in the “market”). (3) Applied data science methods (11)—using machine learning and big data to detect phenotypes of responders and non-responders to treatments and develop personalized treatment protocols. Also, the use of modeling and simulation to optimize stimulation parameters and reduce adverse events or interactions. (4) Network-based approaches (12, 13)—this strategy will be aligned with the multidimensional nature of the pain experience, guiding a new method for developing chronic pain biomarkers that require multimodal and composite assessments (clinical, neuroimaging, and omics). The last two domains can provide the technological advantage and “paradigm change” that would allow to “compete” against dominant treatment strategies. All the papers included in this research topic (two original articles, two reviews, and one case report) can fit in one or more of our proposed domains.

DaSilva et al. proposed and developed a new approach for excitatory stimulation targeting bilateral primary motor cortices that could potentially expand its therapeutic effect to more global pain relief. The authors reported computational models to compare brain current flow for current laboratory-based unilateral and bilateral motor cortex stimulations with a functional home-based prototype. They discussed the promising concept of bilateral excitatory motor cortex stimulation which can be focal and home-based. Considering our framework, this study is related to three of our proposed domains: the use of digital health framework (home-based treatment), bioengineering development (user-centered design of prototype), and applied data science (computational models).

Additionally, Thomas et al. addressed an optimization question of transcutaneous electrical nerve stimulation (TENS) for migraine using computational modeling techniques. The authors developed the first-ever model TENS model for migraine that will support advances, from the exploration of additional electrode designs, considering individual anatomy, and study the extent of variation in current flow. They also found that a extended V-shaped with greater contact separation design has potential advantages but requires further clinical validation.

Similarly, Deblieck et al. used computational models in a different scenario, the safety of concomitant use of more than one neuromodulatory technique. In a 54-year-old woman with an implanted spinal cord stimulation (SCS) system for another pain syndrome, high definition tDCS was initiated for refractory neuropathic pain. The authors reported a notable decrease in pain perception, lasting for approximately 5–6 h with no reported adverse events. The stimulation parameters and clinical efficacy of the SCS system remained unchanged. The computational model indicated no meaningful alteration of current flow. These two studies represent the tremendous utility of computational models (the use of applied data science methods in our framework) for parameters optimization and even to confirm the safety of combined protocols.

Furthermore, Diaz et al. proposed the development of composite pain biomarkers. The authors reviewed current literature on the taxonomy of pain biomarkers and their utilities as pain-related indicators that can help in the diagnosis, treatment, and prognosis of chronic pain. Including physiological non-imaging and imaging biomarkers. Besides, Diaz et al. presented multiple analytic approaches that have been used in the field. The authors highlighted the use of artificial intelligence methods and the integration of multiple types of assessments for the creation of composite biomarkers. This study is an excellent example of the biomarkers optimization for pain neuromodulation using applied data science methods and network-based approaches.

Finally, Castelo-Branco et al. reported interesting insights on two different protocols of pain temporal summation (phasic and tonic) in fibromyalgia subjects, and the associations between these biomarkers and other clinical variables. The result suggests that phasic and tonic protocols are not correlated and could index different neural responses in FM subjects. The authors recommend further studies with larger sample sizes to clarify their results. Likewise, this is an example on how biomarkers optimization is needed to potentially differentiate specific pain phenotypes.

In summary, we believe our proposed four domains and the articles presented here can guide the ongoing optimization of pain neuromodulation. The next generation of neuromodulation devices should therefore provide neuromodulation therapies that could offer rapid acute relief in an easy-to-use manner, thus increasing user's acceptability. The articles presented in this research topic thus provide valuable information and examples of different developmental pathways for optimizing these techniques and harnessing a potentially disruptive innovation highly needed in chronic pain management.
